# Invasive Mucinous Adenocarcinoma Associated with Adjacent Sessile Serrated Lesion of the Appendix Vermiform: A Case Report

**DOI:** 10.1155/2014/979674

**Published:** 2014-07-09

**Authors:** Osamu Kinoshita, Yasutoshi Murayama, Yoshiaki Kuriu, Masayoshi Nakanishi, Chohei Sakakura, Eigo Otsuji

**Affiliations:** Division of Digestive Surgery, Department of Surgery, Kyoto Prefectural University of Medicine, 465 Kajii-cho, Kamigyo-ku, Kyoto 6028566, Japan

## Abstract

Although the definition of sessile serrated lesion (SSL) of colon is controversial and the risk of progression to malignancy is also under investigation at present, SSL is generally described as a polyp characterized by a serrated architecture. It is estimated to represent a feature of a new cancerization pathway, coined “serrated neoplasia pathway,” particularly in right-sided colon adenocarcinomas. On the other hand, in appendix, the role of this pathway remains uncertain, probably because very few cases of appendiceal adenocarcinoma associated with SSL were reported, and furthermore, immunohistochemical examination was rarely carried out. We herein report an interesting case of invasive appendiceal mucinous adenocarcinoma exhibiting SSL, which was pathologically estimated as a potential precursor lesion, and performed representative immunohistochemistry for both the mucinous adenocarcinoma and SSL in the same specimen. To further elucidate the progression of the appendiceal carcinoma from SSL, both an adequate sectioning of the lesion and systematic immunohistochemical examination of a large number of appendiceal carcinoma cases containing adjacent SSL would be required.

## 1. Introduction

Hyperplastic polyp (HP) of colon has been traditionally recognized as a benign lesion; however, recent studies revealed that some types of HP might have a malignant potential, and sessile serrated lesion (SSL) has been increasingly highlighted [1]. Although it has been generally accepted that adenoma-carcinoma sequence [2] is an important pathway in colonic carcinogenesis, recent studies revealed that DNA hypermethylation and/or* BRAF* mutations also play important roles in the tumor progression especially in right-sided colon carcinomas and that SSL morphologically represents a characteristic feature of a newly postulated cancerization pathway, coined “serrated neoplasia pathway” [3, 4]. Moreover, some investigators suggested that neoplastic SSL closely associated with mucinous adenocarcinoma [5], and others reported that the outcome of patients with colorectal carcinomas that featured serrated architecture was worse than that of patients with nonserrated carcinomas [6].

In contrast to accumulative evidence for SSL of colon, SSL of appendix has not yet been fully understood, and the importance of “serrated neoplasia pathway” in appendiceal carcinomas also remains highly controversial [1]. One possible reason is that appendiceal adenocarcinoma exhibiting SSL has been rarely described and the knowledge of immunohistochemistry is limited. Here, we present an interesting case of invasive mucinous adenocarcinoma exhibiting SSL of the appendix, which was estimated as a potential precursor lesion, and describe this case with details of representative immunohistochemical features.

## 2. Case Presentation

A 72-year-old Japanese woman was referred to our hospital for the treatment of a tumor of the ascending colon. The tumor was found by colonoscopy during a checkup for a rectal cancer, which had been resected 5 years earlier, finally staged as pT1 pN0 M0, according to TNM classification of Union for International Cancer Control (UICC). Computed tomography and positron emission tomography revealed that the tumor arose from the appendix and invaded the adjacent ascending colon to expose the mucosal surface. The laboratory data were within the normal range. Because the biopsy from the tumor showed malignancy, ileocecal resection with lymph node dissection was performed. The period after surgery was uneventful, and there was no evidence of recurrence at a 9-month followup, under administration of infusional 5-fluorouracil-based chemotherapy.

**Figure 1:**
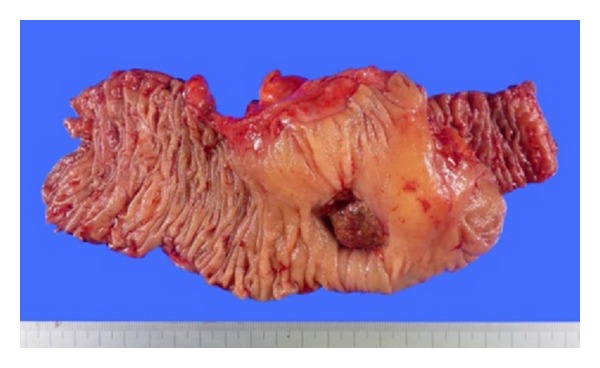
Macroscopic appearance.

**Figure 2:**
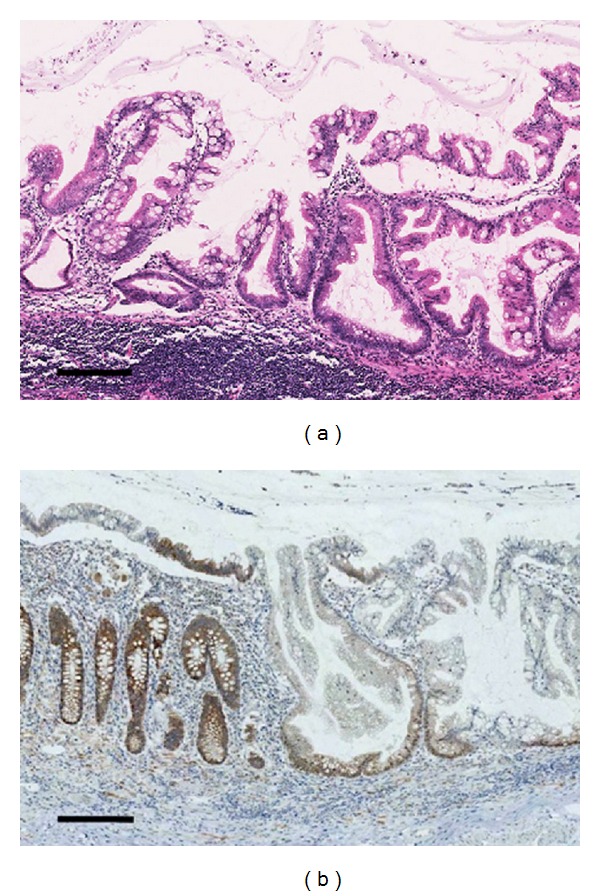
(a) SSL in the root of the appendix. (b) SSL is regionally observed in the right half of the epithelium, while nondysplastic epithelium can be observed in the left half. SSL shows the loss of hMLH-1 expression; however, a weak to moderate reactivity is preserved in the deep part of the crypts. A hMLH-1 positive control was evaluated on nondysplastic epithelium. The scale bars indicate 250 micrometers.

**Figure 3:**
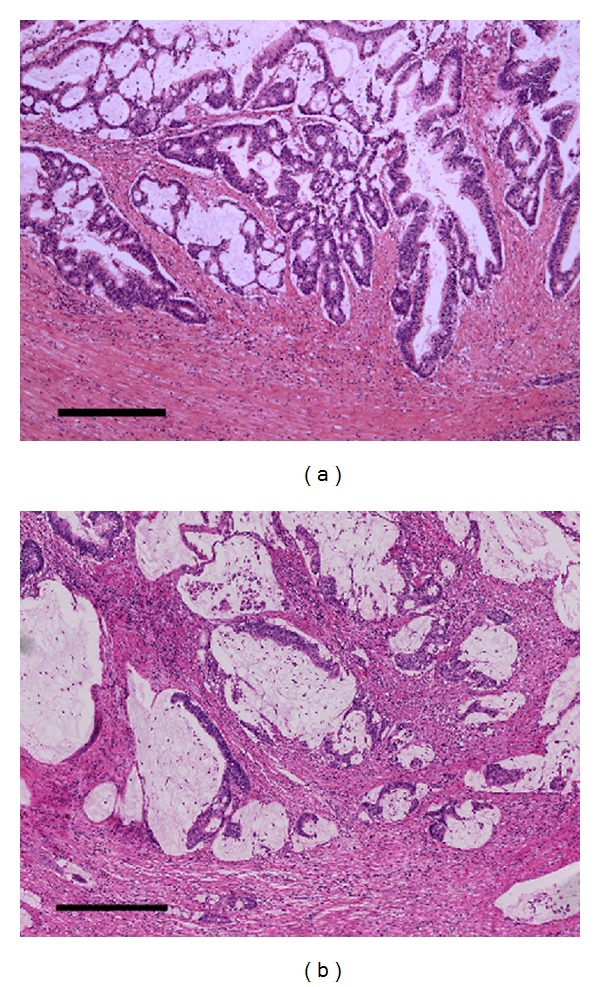
(a) The area of gradual transition between the mucinous adenocarcinoma and SSL in the peripheral side of the appendix, but the serrated structure is not outstanding. (b) The component of mucinous adenocarcinoma without serrated structure. The scale bars indicate 500 micrometers.

Macroscopically, after formalin fixation, the resected specimen showed a clearly demarcated ulcer, measuring 28 × 20 mm maximum dimensions, in the ascending colon (Figure 1). The representative cutting surface revealed that the tumor was mainly located at the peripheral part of the appendix and invaded the adjacent ascending colon to expose the mucosal surface. Pathological findings revealed appendiceal mucinous adenocarcinoma showing extraluminal expansive growth, with one pericolonic metastatic lymph node, finally staged as pT4b pN1 M0, according to UICC TNM classification. The tumor contained massive mucin pools, and well-differentiated adenocarcinomas were found floating in the mucin pools. No goblet cell carcinoid or neuroendocrine cell was observed. The noticeable finding of this present case was the SSL which included serrated structure, crypt dilation, and horizontally arranged basal crypts, in the root of the appendix (Figure 2(a)). Moreover, the SSL combined with dysplastic change extended to the adjacent mucinous adenocarcinoma with an area of gradual transition (Figure 3); this SSL was pathologically regarded as a potential precursor lesion of the adjacent mucinous adenocarcinoma. Immunohistochemical (IHC) analysis was also performed on the formalin-fixed specimens including both mucinous adenocarcinoma and SSL. The experimental conditions, including antibody clone, vendor, and specific dilutions used, are summarized in Table 1. Four-micrometer-thick tissue sections were deparaffinized and heated in a microwave oven, and each immunohistochemistry was performed by using Ventana Benchmark automatic staining system (F. Hoffmann-La Roche Ltd., Basel, Switzerland). Concerning IHC features, the expression of hMLH-1, which is a DNA mismatch-repair protein, was absent in the SSL, although its expression was inclined to be preserved in the deeper part of crypt (Figure 2(b)). On the other hand, the mucinous adenocarcinoma showed none or faint hMLH-1 expression. In particular, p53 protein expression and B-catenin nuclear localization were not observed in both mucinous adenocarcinoma and SSL. Other differences in IHC features are also showed in Table 1.

## 3. Discussion

The words “serrated polyp” were first coined by Jass et al. [7] and were used to describe a lesion with a serrated morphology. However, the gross morphology of SSL resembles that of HP; thus, an obvious distinction between SSL and HP is not entirely easy, even for experienced gastrointestinal pathologists [8]. In addition, whether a SSL without dysplasia can be pathologically recognized as an intraepithelial neoplasia is an unsettled problem [9], and these might be reasons why the definition of SSL has not yet reached consensus amongst pathologists. The current definition of SSL generally applies to a heterogeneous group of lesions characterized morphologically by a serrated architecture [1, 10]. More recently, Fujimori et al. [11] proposed the morphological diagnostic criteria on the basis of their computer assisted cytometrical analysis as follows: (1) crypt dilation, (2) irregularly branching crypts, and (3) horizontally arranged basal crypts (inverted T- and/or L-shaped crypts). Our present case sufficiently fulfilled the criteria above.

We performed a search of the English literature using PubMed with the keywords “appendix (or appendiceal),” “serrated,” and “carcinoma (or cancer)” to search for all case reports of appendiceal carcinoma associated with SSL. To our knowledge, only five cases [9, 12–15] have been published in the past three decades, and most of the studies did not provide IHC analysis comparing both the adenocarcinoma and SSL. Regarding the IHC profile of SSL found in colon, the loss of hMLH-1 and MGMT was reported to play an important role in the serrated neoplasia carcinogenesis pathway [16], and colon carcinomas with high-level microsatellite instability (MSI) have been estimated to be in at least 20% of the right-sided colon carcinomas. On the other hand, although some IHC analysis of appendiceal adenocarcinoma has been reported [14, 17, 18], very few were investigating lesions containing SSL.

A number of authors [9, 19, 20] have previously questioned the importance of DNA mismatch-repair proteins for the development of appendiceal adenocarcinoma. Taggart et al. [19] examined three cases of appendiceal mucinous carcinoma containing SSL and suggested that* MLH1* promoter methylation was not a mechanism for MSI in their series. Likewise, Yantiss et al. [9] described 4 cases of invasive appendiceal adenocarcinoma associated with SSL, and of those, only 1 case showed loss of hMLH-1 expression in both the adenocarcinoma and the adjacent SSL. They suggested that molecular features of the serrated neoplastic pathway were not highly prevalent in adjacent carcinomas. Similarly, in the Japanese literature, Hayashi et al. [14] reviewed 19 cases of appendiceal carcinomas in their respective institutions and described only 1 case associated with SSL, which showed weak expression of hMLH-1 and MGMT in less than 50% of the lesion. Although our IHC results showed the loss of hMLH-1 expression in the accompanied SSL and none or faint hMLH-1 expression in the mucinous adenocarcinoma, the IHC features were almost similar to those in the case previously reported by Yantiss et al. [9]. Moreover, p53 and B-catenin expressions were not observed in both mucinous adenocarcinoma and SSL in our present case. Besides, Ban et al. [21] observed no B-catenin expression in the colonic SSL with dysplasia in their 8 cases, whereas Fujita et al. [5] observed in half of their 12 cases. Concerning mucin expression, Bellizzi et al. [18] examined 53 noninvasive appendiceal epithelial proliferations and suggested that MUC6 expression was associated with SSL morphologic features. However, in our case, we were not able to detect any MUC6 expression in the SSL for unknown reasons.

In summary, the presented SSL pathologically extended to the adjacent mucinous carcinoma with areas of gradual transition; we believe this finding suggested that the tumor potentially arose from SSL of the appendix, and the IHC results were also compatible. This case study was the first to describe the representative immunohistochemistry of both SSL and mucinous adenocarcinoma of the appendix on the same resected specimen. To further elucidate the carcinogenesis and tumor progression from SSL of appendix, an adequate sectioning of the lesion should be performed, and systematic IHC examination of a large number of appendiceal carcinoma cases containing adjacent SSL is required.

## Figures and Tables

**Table 1:** Immunohistochemical features comparing adenocarcinoma and SSL.

Antibody	Clone	Vendor	Dilution	Adenocarcinoma	SSL
p53^a^	DO-7	DAKO	1 : 50	Negative	Negative
MUC2^b^	Ccp58	Leica	1 : 20	Positive	Positive
MUC5A/C^b^	CLH2	Leica	1 : 100	Positive	Positive
MUC6^b^	CLH5	Leica	1 : 100	Negative	Negative
MLH-1^c^	G168-728	Cell Marque	1 : 200	None (or weak)	None
MSH-2^c^	G219-1129	Cell Marque	1 : 1	Strong	Weak
B-catenin^d^	H-102	Santa-Cruz	1 : 200	Negative	Negative

^a^Nuclear staining in more than 10% of the lesion was considered positive.

^b^Any definitive cytoplasmic reactivity was considered positive.

^c^More than 10% of positive staining cell was classified as weak, moderate, and strong, according to the staining intensity.

^d^Aberrant nuclear localization was considered positive.
